# Disorders of sex development (DSD) 46.XY due to type 2 5-α reductase deficiency in three siblings: Case report from a low-resource setting

**DOI:** 10.1016/j.amsu.2022.104577

**Published:** 2022-09-08

**Authors:** Artha Falentin Putri Susilo, Kevin Dominique Tjandraprawira, Patrick Bayu, Hartanto Bayuaji

**Affiliations:** a Department of Obstetrics and Gynaecology, Faculty of Medicine Universitas Padjadjaran – Dr. Hasan Sadikin General Hospital, Bandung, Indonesia; b Department of Obstetrics and Gynaecology, Faculty of Medicine, Universitas Pelita Harapan, Lippo Karawaci, Tangerang, Indonesia

**Keywords:** Disorders of sex development (DSD), 5-α reductase deficiency, Case series

## Abstract

**Introduction and Importance:**

Disorders of Sexual Development (DSD) is a rare autosomal recessive genetic condition significantly affecting patients' lives in various aspects, particularly psychosocially. Type 2 5-α reductase is a cause of DSD 46,XY. It is rare to find multiple DSDs in the same family. Patients may present with amenorrhea and ambiguous genitalia. This case report is aimed to highlight the genetic aspects of the disease, the challenges to diagnostics and the various management options for the patients.

**Methods:**

Case series of three siblings with DSD 46, XY with relevant discussion.

**Outcomes:**

Three sisters, aged nineteen, seventeen, and fifteen years old came with an identical complaint of late menarche. Their physical examinations revealed elementary breast development and little axillary hair. The external genitals consisted of vulva, major and minor labia. Clitoromegaly was present with short (<5 cm) vagina. No female internal genital was found but undescended testes were palpable. Presences of testes was confirmed via ultrasound. Laboratory results showed reduced estradiol, highly increased follicle stimulating hormone (FSH), normal male testosterone levels and increased testosterone-dihydrotestosterone ratio (T/DHT >20). Karyotype was 46,XY. Diagnoses of DSD 46, XY due to type 2 5-α reductase deficiency were established. Patient 1 chose female as the gender of choice whilst patients 2 and 3 chose male. All patients are due for corrective surgery along with psychotherapy and psychoeducation.

**Conclusion:**

DSD 46, XY due to type 2 5-α reductase deficiency is a rare autosomal recessive genetic disorder requiring comprehensive diagnostics and holistic management to improve patient quality of life.

## Highlights

• Disorder of sexual development (46,XY) due to type 2 5-α reductase deficiency is rare.
• It requires a multidisciplinary team involving a battery of tests, imaging and genetic tests, e.g. karyotyping.
• Informed consent and collaboration with other disciplines are pivotal for holistic management.

## 1 Introduction

Disorders of Sexual Development (DSD) is defined as a congenital condition in the form of atypical chromosomal, gonadal, and anatomical reproductive organs development [1]. Such disorders are classified by Lawson Wilkins Pediatric Endocrinology Society (LWPES) and European Society of Pediatric Endocrinology (ESPE) into sexual chromosomal DSD, DSD 46,XX, and DSD 46,XY [2]. Whilst the incidence of DSD is at 1 in 4500 to 5500 newborns, the global incidence of DSD 46, XY is estimated at 1 in 20,000 live births, meanwhile DSD 46, XX is estimated to be 1 in 14,000 to 15,000 live births with congenital adrenal hyperplasia being the most common type [1].

Disorders of Sexual Disorder are diagnosed at different stages of life. Individuals with late puberty, unexplained virilization or gynecomastia, infertility, or gonadal tumor might have a history of ambiguous external genital as a fetus or neonate, gonadal dysgenesis, and mismatch of internal genital with sexual chromosome. This condition might also be found as a part of a genetic syndrome [1].

5-α reductase enzyme is an enzyme that converts testosterone and dihydrotestosterone (DHT). Dihydrotestosterone is a hormone crucial for the development of male genitals. 5-α reductase enzyme consists of two isozyme types: type 1 and type 2 [3]. Type 2 5-α reductase deficiency results in the failure of male genital development among 46, XY patients [4]. As with any disorder, type 2 5-α reductase deficiency exhibits a genetic predisposition. However, it is rare to encounter a case of siblings exhibiting the same disorder. This is a case report three sisters diagnosed with DSD 46, XY due to type 2 5-α reductase in their adulthood, which presented novel complex issues regarding their management and gender identity.

## 2 Case description

The following cases are described according to the PROCESS guideline [5]. This case report registration is on Clinicaltrials.gov with the following identification number: NCT05449080 (URL: https://clinicaltrials.gov/ct2/show/NCT05449080).

### 2.1 Case I

A nineteen-year-old girl presented to the clinic with a chief complaint of late menarche. The patient denied any abdominal pain, cyclic pain, abdominal mass, and vaginal discharge. Patient also denied any disturbance in micturition, constipation, and history of surgical interventions. The patient's two younger sisters also had the same complaint.

**Fig. 1:**
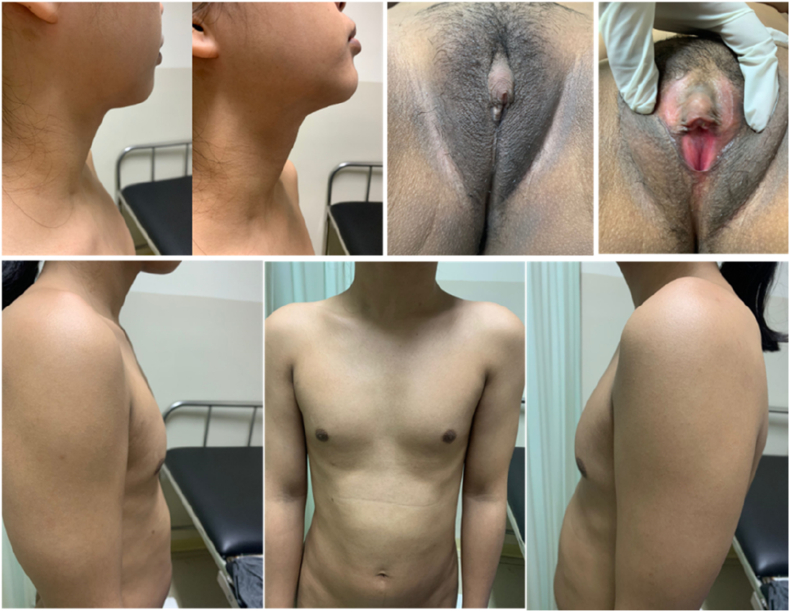
Physical Examination Result of Case 1 (a) Neck examination: no Adam's apple found; (b) external genital: pubic hair development at tanner stage III, 2 cm mass at the anterior vulva; (c) chest examination: breast development at tanner stage I.

Physical examination (Fig. 1) result showed normal vital signs. No Adam's apple was found during neck examination. Chest examination revealed breast development at Tanner stage I and axillary hair development at Tanner stage II. Abdominal examination was unremarkable. External genital examination revealed pubic hair development at Tanner stage III, there was a mass on the anterior vulva in the form of 2 cm phallus, along with major and minor labia within normal limit. The vaginal canal was 1.5 cm in length. Rectal exam revealed only vaginal structure on palpation.

**Fig. 2:**
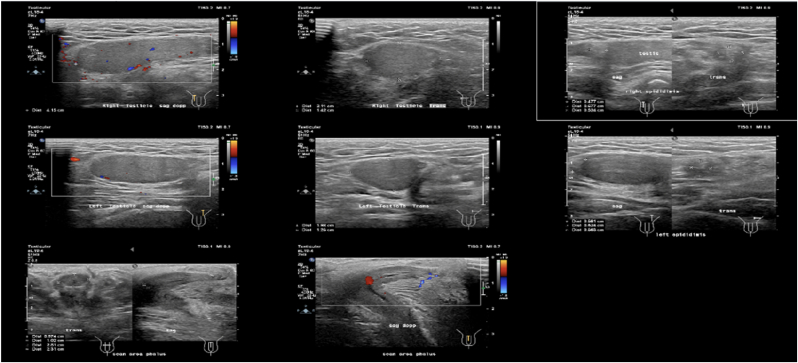
The result of Testicular Ultrasonography.

**Fig. 3:**
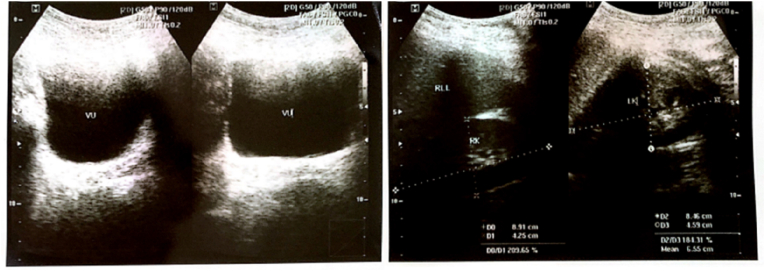
The result of Abdominal Ultrasonography.

Earlier laboratory examination showed reduced estradiol levels (26.35 pg/mL) and highly increased Follicle Stimulating Hormone (FSH) levels (28.94 IU/mL). Blood tests showed normal male testosterone levels (674.4 ng/dL), dihydrotestosterone (14 ng/dL), and Testosterone/Dihydrotestosterone (T/DHT) ratio (48.17). Karyotype revealed 46 XY. Ultrasonography (USG) (Fig. 2, Fig. 3) revealed testis at the right and left proximal inguinal canal, along with phallus at the anterior vulva. Uterus and adnexa were not present on abdominal USG. Pelvic Magnetic Resonance Imaging (MRI) confirmed the structure of bilateral testes, corpus cavernosa, and bulbospongiosus muscle. Uterus and adnexa were not found on MRI. Psychiatric examination on the patient revealed gender dysphoria in adolescent-adult with female as the gender of choice. Surgical correction planned for patients along with psychotherapy and psychoeducation after the surgery.

### 2.2 Case II

A seventeen-year-old girl came to the clinic with a chief complaint of late menarche. The patient denied any abdominal pain, cyclic pain, abdominal mass, and vaginal discharge. Patient also denied any disturbance in micturition, constipation, and history of surgical interventions. This is the younger sister of Case 1.

**Fig. 4:**
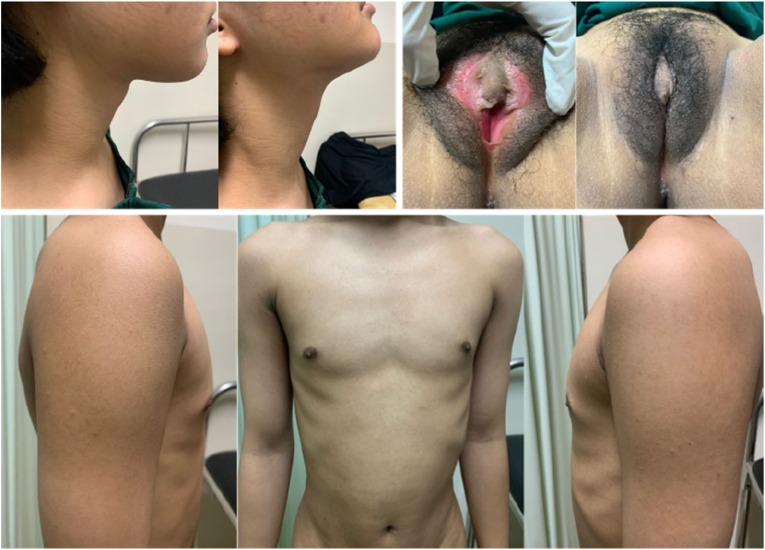
Physical Examination Result of Case 2 (a) Neck examination: no Adam's apple found; (b) external genital: pubic hair development at tanner stage III, hypertrophy of the clitoris, and micropenis; (c) chest examination: breast development at tanner stage I.

Physical examination (Fig. 4) result showed normal vital signs. No Adam's apple was found during neck examination. Chest examination revealed breast development at Tanner stage I and axillary hair development at Tanner stage II. Abdominal examination was unremarkable. External genital examination revealed pubic hair development at Tanner stage III, micropenis, hypertrophy of the clitoris, along with vulva, major and minor labia within normal limit. The vaginal canal was 4 cm in length. Rectal exam revealed only vaginal structure on palpation.

**Fig. 5:**
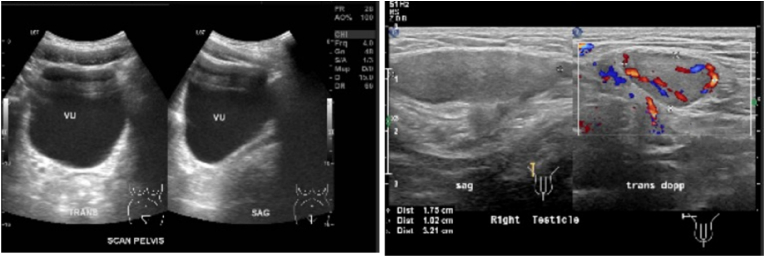
Ultrasonography result: (a) Internal Genitals, (b) Testicular.

**Fig. 6:**
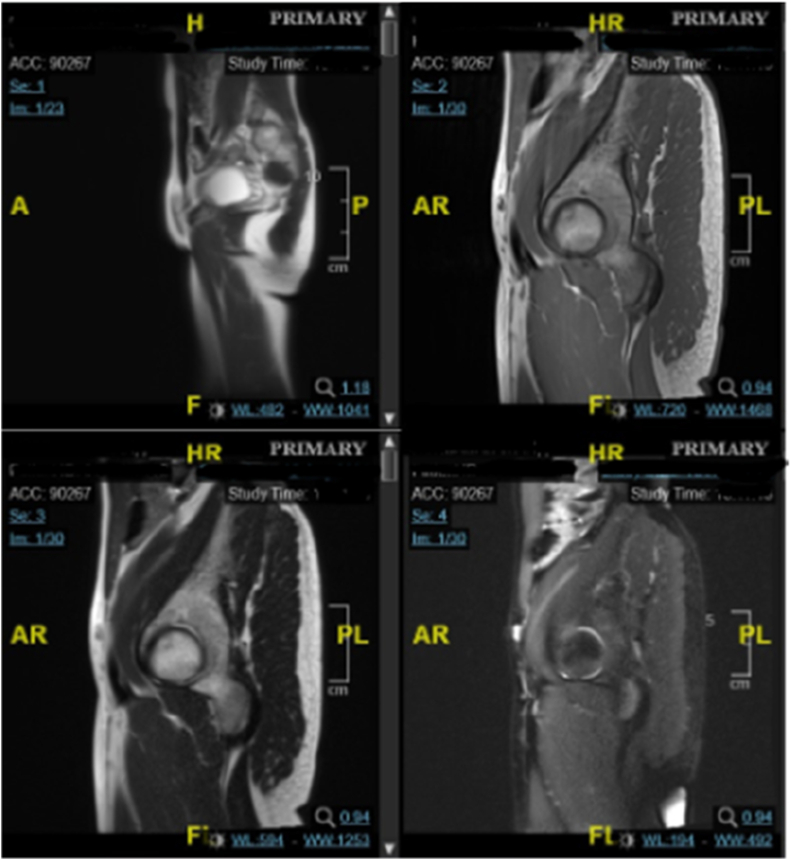
Pelvic MRI result.

Earlier laboratory examination showed testosterone level 271 ng/dL, reduced estradiol level at 23.29 pg/mL and increased FSH at 19.75 IU/mL. The testosterone level was 613 ng/dL, dihydrotestosterone 11 ng/dL, and T/DHT ratio of 55.73. Karyotype revealed 46 XY. Ultrasonography (Fig. 5) examination revealed testis at the right and left proximal inguinal canal, along with phallus at the anterior vulva. Uterus and adnexa were not found on abdominal ultrasonography. Pelvic Magnetic Resonance Imaging (MRI) confirmed the structure of bilateral testes, corpus cavernosa, and bulbospongiosus muscle. Uterus and adnexa were not found on MRI (Fig. 6). Psychiatric examination on the patient revealed gender dysphoria in adolescent-adult with male as gender of choice. Surgical correction planned for patients along with supportive psychotherapy after the surgery.

### 2.3 Case III

A fifteen-year-old girl came to the clinic with the complaint of late menarche. The patient denied any abdominal pain, cyclic pain, abdominal mass, and vaginal discharge. Patient also denied any disturbance in micturition, constipation, and history of surgical interventions. The patient is the younger sister of Cases 1 and 2.

**Fig. 7:**
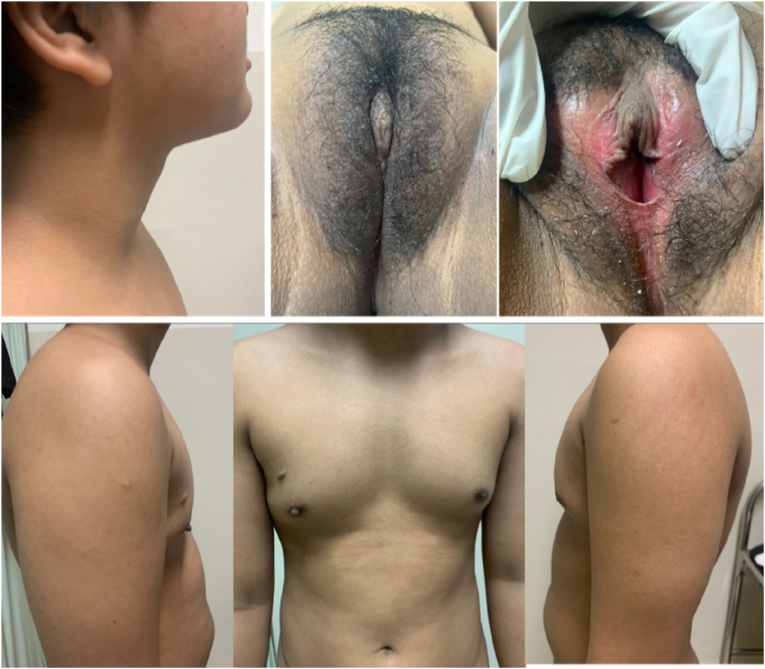
Physical Exam of Case 3 (a) Neck examination: no Adam's apple found; (b) external genital: pubic hair development at tanner stage III, hypertrophy of the clitoris, and 2 cm phallus; (c) chest examination: breast development at tanner stage I.

Physical examination (Fig. 7) result showed normal vital signs. No Adam's apple was found during neck examination. Chest examination showed breast development at Tanner stage I and axillary hair development at Tanner stage I. Abdominal examination was unremarkable. External genital examination revealed pubic hair development at tanner grade III, vulva with clitoral hypertrophy and 2 cm phallus, along major and minor labia within normal limit. The vaginal canal was 1.5 cm in length. Rectal exam revealed only vaginal structure on palpation.

**Fig. 8:**
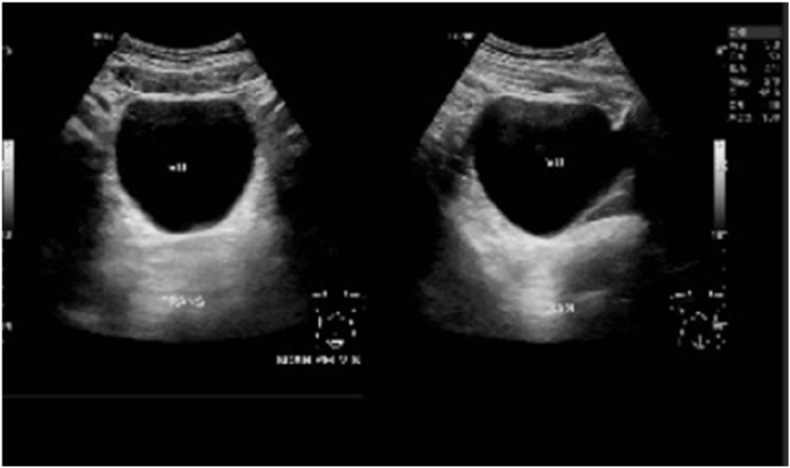
Ultrasonography result.

Earlier laboratory examination revealed heightened FSH (30.45 IU/mL), reduced estradiol (16 pg/mL), testosterone level 466.9 ng/dL, dihydrotestosterone 7 ng/dL, and T/DHT ratio of 66,7. Karyotype revealed 46 XY. Ultrasonography examination (Fig. 8) revealed left testicle outside of the inguinal canal at suprapubic, and right testicle outside of the inguinal canal in the right lower quadrant at the abdomen, along with phallus at the anterior vulva. Uterus and adnexa were not found on abdominal ultrasonography. Pelvic Magnetic Resonance Imaging (MRI) confirmed the structure of bilateral undescended testes, corpus cavernosa, and bulbospongiosus muscle. Uterus, vagina, and cervix were not found on MRI. The patient was diagnosed with DSD 46, XY due to type 2 5-α reductase deficiency, and consultations were urology, pediatric, and pediatric surgery departments were pursued with similar diagnosis as his siblings. Psychiatric examination on the patient revealed gender dysphoria in adolescent-adult with male as gender of choice. Surgical correction planned for patients along with supportive psychotherapy after the surgery.

In summary, the following table compares the attributes of our 3 patients:

**Table:** 

Attributes	Patient 1	Patient 2	Patient 3
Age	19	17	15
Breast development	Tanner stage 1	Tanner stage 1	Tanner stage 1
Axillary hair development	Tanner stage 2	Tanner stage 2	Tanner stage 1
Pubic hair development	Tanner stage III	Tanner stage 3	Tanner stage 3
Genitals	Phallus-like	Clitoromegaly	Clitoromegaly
Vaginal canal	1.5 cm	4	1.5
Karyotype	46 XY	46 XY	46 XY
Ultrasound	Testes (+)	Testes	Testes
MRI	Bilateral testes, corpus cavernosa and bulbospongiosus	Bilateral testes, corpus cavernosa and bulbospongiosus	Bilateral testes, corpus cavernosa and bulbospongiosus
FSH	Increased	Increased	Increased
Testosterone	Normal	Normal	Normal

## 3 Discussion

Type 2 5α-reductase deficiency is an autosomal recessive condition involving the SRD5A2 gene on chromosome 2p23 [6,7]. The enzyme 5 α-reductase is required for the conversion of testosterone (T) to dihydrotestosterone (DHT) through a double-bond reduction with NADPH as a cofactor [8]. Both T and DHT bind to an androgen receptor (AR) and the T-AR and DHT-AR complexes participate in the transcription of factors necessary for sexual differentiation of males [8]. There are 2 isozymes of the enzyme 5 α-reductase: type 1 and type 2 with type 2 isozyme exhibiting a higher affinity towards testosterone [8].

As for the gene SRD5A2, more than 100 mutations have been discovered: 84 involve missense and nonsense mutations spread throughout the length of the gene; 10 alter splicing; 1 found in the regulatory region of the gene; 14 small deletions; 6 small insertion; 3 small indels and 4 large deletions [7,8]. All of these mutations can produce a variety of downstream effects: a complete loss of hormone activity; disrupting receptor binding to testosterone; diminished NADPH activity; impairing the assembly of hormone-receptor complexes; and decreasing the half-life of hormones [8]. As a consequence, the phenotype of type 2 5α-reductase deficiency is wide-ranging, attributed to the varying degrees of residual enzymatic activity and also, the underlying genetic abnormality [8].

In addition to the mutations, there have also been polymorphisms, further convoluting the genotype-phenotype relationship [7]. The most studied is the V89L polymorphism, in which a valine is replaced by a leucine at codon 89. This is more commonly encountered amongst Indian and Chinese populations and increases the risk for hypospadia [8].

It is commonly believed that there is little to no genotype-phenotype correlation among those with the same phenotype. Avendaño et al. conducted a study in which 256 patients were reviewed and to each patient, an external masculinization score (EMS) was assigned [8]. The score ranged from 0 to 12 with 12 being a normal male phenotype. They divided the mutations among their patients as mutations affecting testosterone activity, NADPH binding activity and enzyme activity respectively. They managed to show that mutations in each group produced different phenotypes and ranges of EMS. It was observed that when the genotypes impairing testosterone activity produced the lowest EMS and the most female phenotype whilst those affecting the enzyme activity produced the least severe phenotypes. To summarise, even though there is no strong genotype-phenotype correlation, it is worth examining, if possible, the exact mutation in each patient as it may explain the patient's phenotype.

Marzuki et al. provided an excellent study on the variety of SRD5A2 gene variants among Indonesian patients with this disorder [6]. They discovered that among 37 subjects, there were twelve variants of the SRD5A2 identified and 6 of which were novel. They also agreed with Avedaño et al. in which the mutations themselves produce deficiency at different levels of severity [6]. More severe diseases produce subjects with lower EMS. They also concluded that there is little genotype-phenotype correlation whilst retaining the merit for specific genotypic diagnosis of the disorder [6].

As described above, the phenotype varies widely, as is the case of our three siblings. In our case series, all three subjects had normal looking vulva with corresponding labia majora and minora. Thus, they were raised as females. However, once they reached puberty, the vulvar enlargement in case 2 formed a micropenis, meanwhile cases 1 and 3 formed phalluses.

Imaging revealed no female internal genital structure and only undescended testes. Surgical correction on DSD 46, XY is generally recommended for male rearing in order to maintain feritlity potential. However, the decision for gender on DSD patients should be made by the patient and the family. Psychological examinations could help make a more holistic decision [9].

Unfortunately, cases of multiple siblings affected by the same disorder have been reported elseware. Type 2 5α-reductase deficiency on three brothers has also been reported in India [10]. The phenotype on the first and second brothers were in the form of penoscrotal hypospadia and micropenis, meanwhile the third brother was in the form of bifid scrotum. This resulted in the patients being raised as males [10].

Ideally, a psychological evaluation must be performed before 27 months of age to avoid identity conflicts [8]. Frequently, exposure to androgens around puberty causes the development of male gender identity in adolescence or early adulthood [8]. This is encountered in our patients too. They had been raised as females in their childhood but come puberty, gender identity conflicts arose due to masculinizing effects of testosterone. Unfortunately, lack of awareness led to a delayed recognition of this disorder and the plan for sex reassignment only in late adolescence and/or early adulthood. Psychological evaluation was performed on our patients and patients 2 and 3 chose male as the gender of choice, hence the decision for masculinizing genitoplasty.

A delayed diagnosis leading to management only during early adulthood has been reported elsewhere. Maleki et al. described managing a 21-year old type 2 5α-reductase deficient patient choosing to be a female [11]. She underwent bilateral gonadectomy, recessive cliteroplasty, urethroplasty and vaginoplasty and received hormonal replacement therapy (HRT) using low dose estrogen to develop the female sexual characteristics.

In addition to surgical treatment, hormonal therapy may be indicated as required. Among patients choosing to be males, testosterone replacement is generally not required as they still retain testicular function during puberty [8]. However, high doses of testosterone or dihydrotestosterone may be prescribed to improve male secondary sexual characteristics, in particularly body hair and penile length [8]. Among patients choosing to be females, such as case 1, hormonal therapy is required to develop female secondary sexual characteristics. Low estrogen doses should be prescribed to allow normal breast development and maintained continuously [8]. Vaginal dilation should also be performed, often with acrylic molds, for those wishing to be active sexually.

There is no exact consensus with regards to the time of surgical correction, whilst noting the importance of multidisciplinary approach for the treatment [12]. Whilst certainly out of this case report's review scope, generally the surgical correction for patients who choose male as the gender of choice would consist of hypospadias correction, such as chordee correction, orchidopexy, and urethra reconstruction [13,14]. Masculinizing genitoplasty in DSD patients is ideally performed within the first 18 months of life.

Patient 1 chose female as the gender of choice hence arrangements were made for feminizing genitoplasty. Surgical correction would consist of increased vaginal canal size to the perineum and vaginal and urethral separation. Clitoral reduction is considered in cases with heavy virilization and performed along with urogenital sinus correction. Feminizing genitoplasty is recommended within the first year of life but vaginoplasty is usually performed during adolescent [13,14].

## 4 Conclusion

Type 2 5α-reductase deficiency is a rare genetic condition causing Disorders of Sex Development (DSD) 46,XY. This condition will hugely affect the lives of patients suffering from it. The case of three sisters in this case series showed that the management of each cases should be tailored according to the need of the patients. Holistic diagnosis and management could help improve the quality of life of the patients.

## Ethical approval

This study does not require an ethical approval as determined by the institutional and departmental review board.

## Sources of funding

The study did not receive external funding.

## Author contribution

AFPS, KDT, PB, and HB conceived the study. AFPS was the lead consultant of the patients. AFPS and PB were responsible for collecting patient data. AFPS and KDT drafted the manuscript. All authors reviewed the manuscript and have agreed this final form of manuscript for publication.

## Trial register number

This case report registration is on Clinicaltrials.gov with the following identification number: NCT05449080 (URL: https://clinicaltrials.gov/ct2/show/NCT05449080).

## Guarantor

The guarantor of this study is Artha Falentin Putri Susilo, M.D.

## Consent

Written informed consent was obtained from the patients for publication of this case report and accompanying images. A copy of the written consent is available for review by the Editor-in-Chief of this journal on request.

## Provenance and peer review

Not commissioned, externally peer-reviewed.

## Declaration of competing interest

The authors declare that we have no conflicts of interest.
